# The Rubber Question

**Published:** 1868-06

**Authors:** 


*The Rubber Question.* Almost daily for a month past, we have been in receipt of letters asking for advice upon this subject, and we propose in a brief manner to inform our readers how the matter stands at the present time.

A few weeks ago the publishers of the Journal received a letter from the Porter Manufacturing Company. (Agents for the Simpson rubber under the Hale patent) stating that in the case of the Goodyear Company against Dr. Evans, a preliminary injunction had been granted by the U. S. District Court of New York, against the defendant, owing to the defence not being properly attended to.

The letter also states that Mr. Hale at the present time is using all his energy to defend the patent, in a case to be tried in Hartford Conn.

The lawyers for the defence have advised Mr. Hale to make this case in Hartford a test one, and to let the others be governed by it.

Many of the Dentists of Baltimore have acceded to the demands of the Goodyear Company and taken out licenses for one year from Jan. 1st, 1868. They have been governed in a great measure by the result of the New York case, and also by the fact that injunctions are readily obtained in the U. S. District Court of Maryland, the cost of which to the defendant amounts to a sum which will procure a license for one year from the Vulcanite Company. They also regard this yearly license as being less objectionable, than a tax on each set made.

In Cincinnati the Dentists are still contending against the demands of the Goodyear Company and with some hope of success. In the case of the Goodyear Company against Dr. Berry in the U. S. Southern District Court of Ohio, no decision has yet been rendered, it apparently being a more difficult matter for the Vulcanite Company to obtain injunctions in Cincinnati, against the Dentists claiming to use the Simpson rubber, than in Baltimore.

In the argument of S. S. Fisher Esq. Counsel for defendant in the Ohio case, the following points are urged: I. The two reissued patents upon which this suit is brought are void, because they are not for the same invention as the original." II. The reissues were improperly granted." "III. Infringement is not proved." "IV. The open and notorious purchase and use of soft rubber by the Dentists of this vicinity, for the purpose of making vulcanized plates, is a bar to a proceeding in equity for an injunction. The only remedy of complainants, if they have any, is at law."

In the case decided recently in New York the Court held: "That on the affidavits of experts, the article made under the Simpson patent, before analysis, has the properties of the Nelson Goodyear hard rubber, and the analysis of it shows that it contains not less than four ounces of sulphur to a pound of rubber, and nothing more is needed to show that the use of it, and the manufacture of it, are infringements upon the plantiffs patent." "An injunction must be issued as prayed for."

We shall advise our readers of the result of the Hartford case when the trial takes place.

